# Ectopic Cervical Pregnancy: Treatment Route

**DOI:** 10.3390/medicina56060293

**Published:** 2020-06-12

**Authors:** Guglielmo Stabile, Francesco Paolo Mangino, Federico Romano, Giulia Zinicola, Giuseppe Ricci

**Affiliations:** 1 Institute for Maternal and Child Health IRCCS “Burlo Garofolo”, 34100 Trieste, Italy; francesco.mangino@burlo.trieste.it (F.P.M.); federico.romano@burlo.trieste.it (F.R.); giuseppe.ricci@burlo.trieste.it (G.R.); 2 Department of Medicine, Surgery and Health Sciences, University of Trieste, 34100 Trieste, Italy; giulia.zinicola@burlo.trieste.it

**Keywords:** cervical pregnancy, fertility-sparing treatment, hysteroscopy, methotrexate, ectopic pregnancy

## Abstract

*Background and objectives:* Cervical pregnancy (CP) is a rare form of ectopic pregnancy (EP) in which the embryo implants and grows inside the endocervical canal. Early diagnosis is essential in order to allow conservative medical and surgical treatments. Although many treatment approaches are disponible, the most effective is still unclear. The aim of this study is to evaluate the efficacy of hysteroscopic management in early CP in order to preserve future fertility. *Materials and Methods:* This is a retrospective observational case series. Five patients with a diagnosis of CP, hemodynamically stables and managed conservatively between 2014 and 2019 at the Institute of Child and Maternal Health Burlo Garofolo in Trieste, Italy, were included. Four patients, with βhCG levels > 5000 mUi/mL were managed by hysteroscopy, with or without a previous systemic Methotrexate (MTX). One case with βhCG levels < 5000 mUi/mL was treated using MTX combined to Mifepristone and Misoprostol. *Results:* In one patient treated by hysteroscopy alone it occurred a profuse vaginal bleeding with necessity for blood transfusion. Haemorrhage was controlled by a second hysteroscopic procedure. No complications, such as vaginal bleeding, were recorded in the other cases. Serum β-hCG levels become undetectable in a range of 15–40 days after hysteroscopic management; after medical treatment it become undetectable after 35 days. Serum βhCG levels had a faster drop the day after hysteroscopy than post medical management. The onset of a spontaneous pregnancy at the normal implantation site occurred after five months in one case treated by hysteroscopy. *Conclusions:* Many therapeutic approaches are effective for CP treatment. Hysteroscopy, alone or in combination with MTX, may provide a greater effect on the descent of βhCG, leading to a reduction of the hospitalization stay, decreasing costs and period for attempt pregnancy. Further prospective studies on larger samples are needed to define therapeutic protocols for CP management.

## 1. Introduction

Cervical pregnancy (CP) is a rare form of ectopic pregnancy (EP) in which the embryo implants and grows inside the endocervical canal. Non-tubal EPs account for less than 10% of all EPs, though their overall incidence has been increasing in recent years [1]. CP incidence ranging between one in 1000 and one in 18,000 of all pregnancies and 1% of all EP [2]. Although the aetiology of CP is unknown, a history of dilation and curettage in a previous pregnancy has been identified as risk factors in nearly 70% of cases [1]. Other predisposing factors are previous caesarean delivery, cervical surgery, endometritis, use of an intrauterine device and in-vitro fertilization (IVF) [3]. Diagnosis involves a combination of clinical symptoms, serology, and ultrasound. The most common symptom of CP is vaginal bleeding, which is often profuse and painless [4]. Other clinical signs can be found at physical examination, such as a softened and disproportionately enlarged cervix due to the product of conception confined within the endocervix and a closed internal uterine orifice (os) with a partially open external os. On speculum examination, the typical signs of CP are represented by a bulky cervix with bluish mucosa and dilated and tortuous submucous vessels. Clinical recognition of CP remains difficult. If diagnosis is delayed, heavy vaginal bleeding and acute abdominal/pelvic pain can occur. Serial βhCG levels is commonly used to monitor early pregnancies but the ultrasound findings of the gestational sac (GS) is essential. CP is identified on trans-vaginal ultrasounds (TVUS) by a distended cervical canal containing a GS with peripheral Doppler flow, below a closed internal cervical os. The ‘sliding organ’ sign, should be absent in a CP [5], while it is associated with spontaneous abortions in progress [1]. Pelvic magnetic resonance imaging (MRI) could be helpful, as supplementary, when the diagnosis is uncertain [4]. CP-related hemorrhage could be life-threatening and frequently necessitates a hysterectomy. Early diagnosis is essential in order to allow conservative medical and surgical treatments. Although many treatment approaches are disponible, the most effective is still unclear. Medical management, using local or systemic Methotrexate (MTX), is a safe and effective option in most clinically stable patients. It has been considered in the conservative EP management because it blocks the trophoblast cell division and inhibits trophoblast proliferation. Hysteroscopic CP resection has been reported as a feasible conservative strategy. Medical and surgical techniques are frequently used in combination [6]. We present a case series of four early gestational CPs managed by hysteroscopy alone or in combination to systemic MTX and one case treated solely medically in order to preserve future fertility.

## 2. Materials and Methods

Women diagnosed with CP between 2014 and 2019 at the Institute of Child and Maternal Health Burlo Garofolo in Trieste, Italy, were included in this case series report. Only cases of CP in stable patients managed conservatively were included. Written informed consent was obtained from all individual participants before procedures. Permission for the publication was taken in accordance with the 1964 Helsinki Declaration and its later amendments or comparable ethical standards (the institutional code number approval is RC 02/2020 from February 2020). Obstetrical/gynecological history, previous risk factors for CP, serum βhCG levels at the diagnosis, ultrasounds findings, surgical or medical management, treatment outcomes were presented. Diagnosis of CP was made by TVUS according to criteria reported by Hoffman [7] and they consist of: No evidence of intrauterine pregnancy, hourglass shape of uterus, cervical ballooning, presence of placental tissue or gestational sac within the cervical canal and closed internal os. Searched for also was the ‘sliding sign’ to exclude ongoing intrauterine pregnancy abortion as described by Jurkovic [5]. Serial βhCG levels were used to monitor CPs before and after treatment since it was no longer detectable (<5 mUI/mL). Fertility-sparing treatment options including medical therapy and hysteroscopic removal of the CP were discussed with the patients. The risk of profuse haemorrhage during hysteroscopy and the following possible need for emergency hysterectomy was also explained, written and signed in a consent form. The size of the GS did not guide in treatment choice because the diameter of the pregnancies was almost similar. Conversely, we thought that high βhCG levels could be related to a larger trophoblast. Therefore, the size of the trophoblast might be directly related to a greater failure of medical therapy and a greater risk of bleeding. For this reason, we considered βhCG levels as an indirect sign of trophoblast size that allowed to stratify patient’s risk of bleeding and to personalize the therapeutic approach. In one case, a total medical management using a single dose of Methotrexate IM (intramuscular) 50 mg/m^2^ of the body surface in addition to Mifepristone 600 mg and Misoprostol 400 mcg orally was opted for. Two patients were totally treated by hysteroscopy; two patients had a previous single MTX IM injection at dosage of 50 mg/m^2^ of the body surface followed by hysteroscopy. A 5 mm Bettocchi hysteroscope (Storz^®^) with 5 Fr bipolar electrode Versapoint Twizzle (Gynecare^®^, Johnson & Johnson–Ethicon Endo-Surgery, Inc., Cincinnati, OH, USA) was used in order to identify and have access to the GS, cervix was dilated and a resectoscopy was made; with a 10 mm resectoscope with a bipolar electrode Versapoint (Gynecare^®^) was obtained a complete resection of the residual chorial villi. Bipolar coagulation was used to reassure hemostasis. In one out of four cases, an Intracervical (IC) Foley catheter n14 filled with 50 mL of saline solution with haemostatic function was inserted. 

## 3. Results

### 3.1. Population

Patients’ obstetrics/gynecological history, risk factors for CP, laboratory, and treatment results are presented in Table 1.

The age range at the moment of diagnosis was between 35 and 41 years old. One of the patient had a history of recurrent spontaneous abortion in particular she reports multiple curettages for termination of pregnancy twice and a previous CP treated in another center by double dose of MTX IM 50 mg/m^2^ of body surface and followed by RCU of the endometrial cavity; another one had a history of tubal EP with spontaneous resolution. One patient had a single spontaneous miscarriage in the 1st trimester of pregnancy. Only in one case risk factors for CP were not identified.

Two patients conceived spontaneously and the other three conceived after IVF-ET. Gestational ages (GA) was up to six weeks and six days in four patients which received surgical management as the sole treatment (*n* = 2) or in addition to a single MTX IM injection at the dosage of 50 mg/m^2^ of the body surface (*n* = 2); one patient was at 10 weeks and four days of amenorrhea but six weeks at ultrasound evaluation and she had a total medical management and her serum β-hCG levels at the moment of the diagnosis was <5000 mUi/mL. Patients treated by hysteroscopy as the sole strategy (*n* = 2) had high serum β-hCG levels at the moment of the surgical procedure, respectively 55,951 mUi/mL and 10,861 mUi/mL; conversely, the other three cases had value serum β-hCG < 10,000 mUi/mL.

### 3.2. Interventions

**Figure 1:**
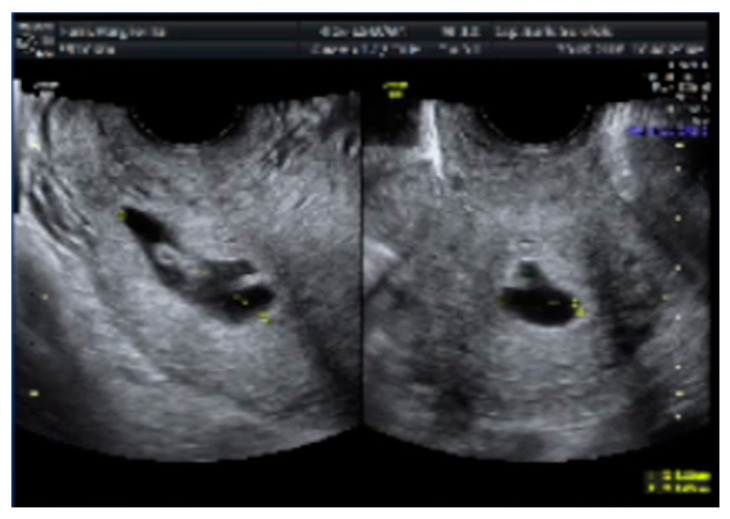
Ultrasound images of cervical pregnancy. (**left**) A gestational sac in the cervical portion of the uterus with embryo echoes; (**right**) A distended cervical canal containing the gestational sac.

**Figure 2:**
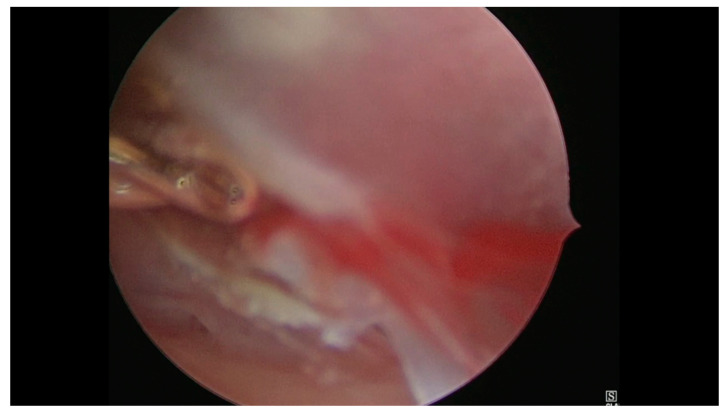
Hysteroscopic view of the gestational sac section.

**Figure 3:**
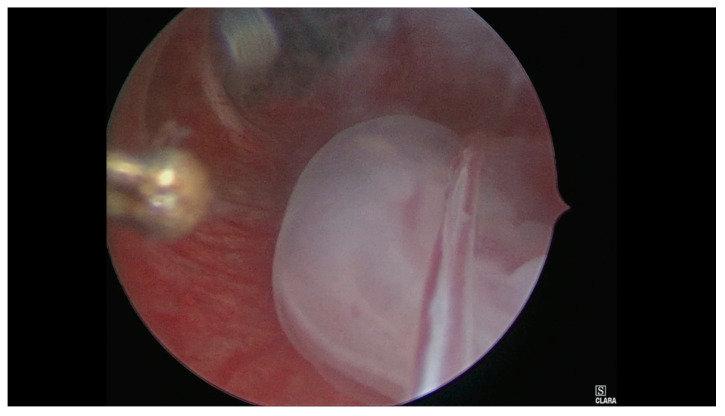
Hysteroscopic view of the embryo.

**Figure 4:**
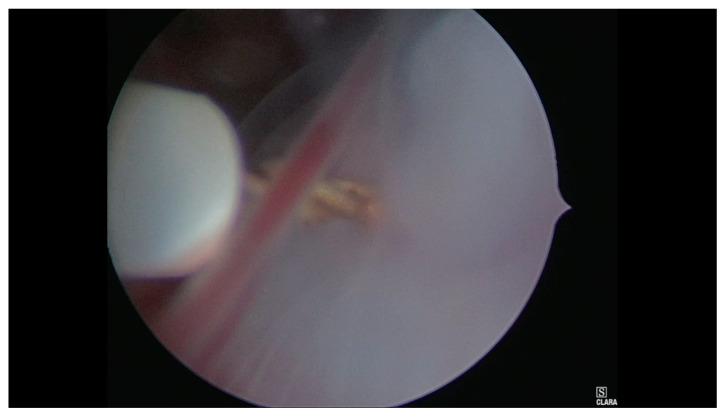
Hysteroscopic view of the umbilical cord section.

**Figure 5:**
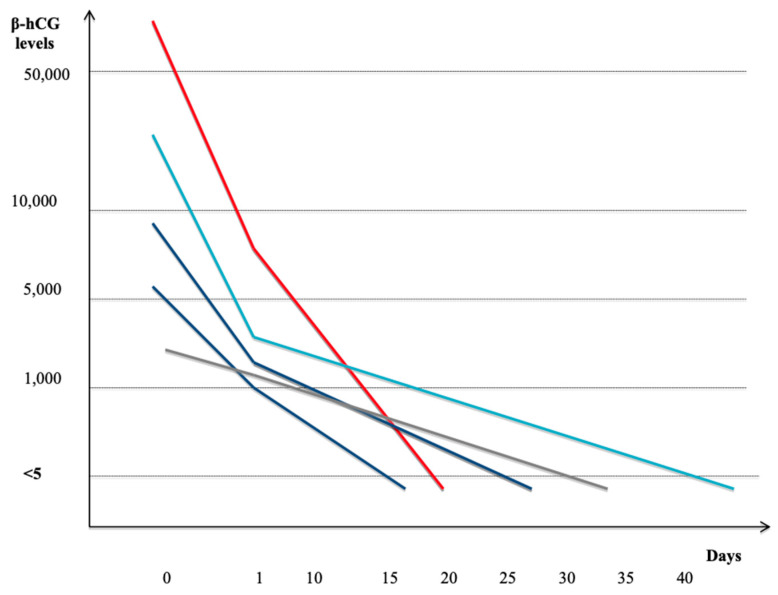
Serum β-hCG levels after fertility-sparing treatment of cervical pregnancy. Total hysteroscopic management: complete resection of CP and coagulation of implantation site (red); Total hysteroscopic management: interruption of CP and coagulation of vessels (light blue); MTX IM 50 mg/m^2^ and hysteroscopy (blue); Medical management: MTX IM 50 mg/m^2+^ Mifepristone 400 mcg orally + Misoprostol 600 mg orally (gray).

- Case 1. Hysteroscopy was performed in two steps. During the first phase a 5 mm Bettocchi hysteroscope (Storz^®^, Karl Storz SE & Co, Tuttlingen, Germany) with 5 Fr bipolar electrode Versapoint Twizzle (Gynecare^®^) was used to identify the GS: it was opened and the pregnancy terminated by cord section and vessels were partially coagulated; subsequently, the cervix was dilated and we performed a resectoscopy. During the second phase the GS and the embryo were removed and a 10 mm resectoscope with bipolar Versapoint (Gynecare^®^) was used to obtain a complete resection of the residual chorial villi. Lastly, we performed an electrocoagulation of the bleeding vessels on implantation site, in order to control the hemostasis (Figure 1, Figure 2, Figure 3 and Figure 4)
- Case 2. Hysteroscopy allows direct visualization of the CP whom partial resection was made by a 5 Fr bipolar electrode Versapoint Twizzle (Gynecare^®^). An IC Foley catheter n14 filled with 50 mL of saline solution with hemostatic function was inserted. Due to persistent vaginal bleeding, the patient was submitted to a second operative hysteroscopy with the aim of removing persistent trophoblastic material and stopping bleeding in the site of its implantations through electrocoagulation by a 10 mm resectoscope with bipolar electrode Versapoint (Gynecare^®^)
- Case 3. Because of the initial serum β-hCG level (1100 mUi/mL) and a desire for future pregnancies and a less invasive procedure, one systemic dose of MTX IM 50 mg/m^2^ of body surface was administrated. Despite that serum β-hCG level raised to 5074 mUi/mL in a week. So we arranged for a hysteroscopic treatment of the CP by a 5 Fr bipolar electrode Versapoint Twizzle (Gynecare^®^).
- Case 4. On the basis of the previous experience and the β-hCG level (9747 mUi/mL), a single systemic dose of MTX IM 50 mg/m^2^ of body surface was administrated, followed by the hysteroscopic CP interruption by a 5 Fr bipolar electrode Versapoint Twizzle (Gynecare^®^).
- Case 5. A total medical management was chosen considering the 10 weeks of amenorrhea but the six weeks of GA at ultrasound evaluation. Furthermore, the βhCG level of 1331 mUi/mL led us to use a single dose of MTX IM 50 mg/m^2^ of the body surface in addition to Mifepristone 600 mg and Misoprostol 400 mcg orally (Table 1; Figure 5).

### 3.3. Outcomes

Nor suction neither curettage were performed in any cases for evacuation of gestational products, before or after hysteroscopic detachment of the GC. In one of the total hysteroscopic case procedure was interrupted before complete resection of the pregnancy. A profuse hemorrhage led to a blood loss of 1400 cc and the patient underwent a blood transfusion. A second hysteroscopy with the aim of remove CP completely was administrated the following day (Table 1). No uterine artery embolization (UAE) was performed to prevent hemorrhage and control hemostasis. No complications, such as vaginal bleeding, were recorded at the end of hysteroscopic procedure after complete CP resection and coagulation of the vessels in implantation site. 

Serum βhCG levels decreased faster since the day after the procedure and it became undetectable in a range of 15–40 days after hysteroscopic management. The patient who received only the medical treatment had a serum βhCG level undetectable in 35 days (Figure 5) but TVUS findings showed the presence of a persistent vascularized lesion in correspondence of the implantation site after five months.

## 4. Discussion

Currently no therapeutic protocols have yet been established worldwide because CP management experiences are based mainly on case series studies due to the rarity of the condition [8]. The choice of treatment depends on GA, initial serum βhCG level, fetal heartbeat presence, vaginal bleeding and desire to preserve fertility. Advanced GA frequently is associated to high morbidity and greater risk of hysterectomy in case of CP [3]. Many studies showed that MTX is safe and effective in CP treatment, but which dose, schedule and follow-up protocols are yet to be determined [9]. Advanced GA (>9 weeks), high serum βhCG levels (>5000 mUi/mL) and presence of a viable embryo (with a crown rump length (CRL) > 10 mm) are variables associated with higher rates of treatment failure [9]. 

A single systemic dose of MTX 50–75 mg/m^2^ is widely accepted as the first-line therapy regardless of fetal cardiac activity. It is important to keep in mind that MTX, especially systemically administrated, could be associated to some side effects such as nausea, upset stomach, diarrhea, stomatitis, fever, headache, fatigue, hepatotoxicity, and myelosuppression. In addition, monitoring patients during βhCG negativization is essential, and a long hospital stay after MTX therapeutic protocols is not unusual. Moreover, in approximately one third of cases additional surgical procedures are needed to reassure and/or complete the treatment of a CPs [6]. Sexton and Sharp in 2002 had reported the first clinical description of a CP successfully treated with MTX and Mifepristone. The exact mechanism of action of this two-drug combination is unclear. It has been suggested that MTX anti-trophoblastic effect is enhanced by Mifepristone antidecidual-like action, with the subsequent cervical trophoblast destruction [10]. The association of Misoprostol appears to be useful to aid cervical evacuation by increasing the frequency and intensity of uterine smooth muscle contractions, speeding up the resolution process [10]. According to current literature in one patient (βhCG < 5000 mUi/mL) we chose for complete medical treatment taking advantage of the possible synergistic action of the two drugs, MTX and Mifepristone, and the Misoprostol action in promoting CP evacuation. (Table 1 and Table 2) Patients had a long follow up by ultrasounds and laboratory testing, without any complications. Complete resolution had been testified by serum βhCG negativization (Figure 5); however, TVUS showed an image due to residual CP in the cervical portion after four months. Some authors have considered UAE to be the only alternative to hysterectomy for bleeding control in case of CP. It plays an important role in the conservative management of some gynecological hemorrhage by causing an extensive blockage of the uterine vascularity [8]. Anyway, re-bleeding or delayed bleeding can occur after UAE, because the extensive collateral circulation to the cervix will be established within hours [11]. Curettage is a quick approach to evacuate conceptional tissue from the cervix. However, in cases of CP, the gestational tissue may implant deeply into the cervix, making it so difficult to achieve optimum evacuation by using curettage, consequently increasing the risk of re-bleeding [11]. After uterine arteries occlusion there would be no significant bleeding during curettage [12]. It is important to remind that UAE could have various complications. These include endometrial atrophy leading to secondary amenorrhea and uterine necrosis. UAE could be responsible of the altered reproductive outcomes due to the related compromised ovarian function and premature ovarian failure in some cases [13]. The exact incidence of uterine necrosis after UAE is difficult to ascertain with only a handful of cases reported in literature [13]. 

In our opinion, other strategies could be managed in order to preserve future fertility. Hysteroscopy for early first trimester CP is a potentially safe and effective option for fertility-sparing management after failure or in addition to MTX [6,14]. It allows direct visualization of the cervical canal and uterine cavity and, most of the time, it is a well-tolerated procedure, used also in combination with UAE [15]. The depth and extent of embryo implantation on the cervical wall is unpredictable but it seems that the earlier the gestation occurring, especially when less than 8 weeks, the easier and freer of complications the hysteroscopic management. Operative hysteroscopy may be also performed with the purpose of obtain a complete resection of GS in order to prevent tissue retention, which can cause serious complications such as persistent bleeding and infection. The complete trophoblast removal under direct visualization may prevent vaginal bleeding and it is followed by the rapid decline of the βhCG level. This mini-invasive surgical procedure, whose satisfactory response is testified by the βhCG trend, can reduces the hospitalization stay of the patient with an abatement of costs and a reduction in the time for the onset of a future pregnancy [16]. In two of our cases we decided firstly for a systemic medical management with MTX. Patients had any symptoms at the moment of the diagnosis, while βhCG levels were between 5.000 and 10.000 mUi/mL. To prevent bleeding enhancement and considering desire for future pregnancies in a short time, we thought that a good option could be to combine medical treatment and hysteroscopy using a 5 Fr bipolar electrode twizzle (Table 1 and Table 2). Any post-procedure complications were recorded, βhCG levels became negative in a short time (20–25 days) (Figure 5). In two cases we decided to perform a total hysteroscopic approach: patients had any symptoms at the moment of the diagnosis, while βhCG levels were >10.000 mUi/mL. In one case during hysteroscopy (Table 2), pregnancy was interrupted using twizzle and it appeared heavy vaginal bleeding. An IC Foley catheter n14 filled with 50 mL of saline solution was placed with hemostatic function. Blood loss was 1400 cc and the patient had a blood transfusion. In spite of the continous slight vaginal bleeding, the patient was submitted to a second hysteroscopy with the aim of controlling bleeding: trophoblastic residuals were removed and vessels in the site of implantation were coagulated by a 10 mm resectoscope (Gynecare^®^). This suggested to us that treating CP hysteroscopically could be a safe option if it is possible, performing the pregnancy interruption and the resection of trophoblastic residuals, with a prompt coagulation of the vessels in correspondence of implantation site [17]. On the basis of our previous experience, the two steps technique (Bettocchi hysteroscope with Twizzle followed by Resectoscope) was used and no complications were recorded after procedure, with a fast βhCG reduction (Figure 5) and no evidence of residual CP images during ultrasounds follow-up. The onset of a spontaneous pregnancy at the normal implantation site occurred after five months. In our opinion, according to the present literature [18], the hysteroscopic approach is a safer, faster, and more accurate technique when performed by an expert surgeon in order to obtain a complete clinical resolution, control hemostasis and avoid hysterectomy. This technique, in comparison with other methods such as curettage and UAE, provides a direct visualization, a precise resection and coagulation of the ectopic tissue, achieving complete eradication with minimal bleeding and preservation of reproductive activity. 

## 5. Conclusions

**Figure 6:**
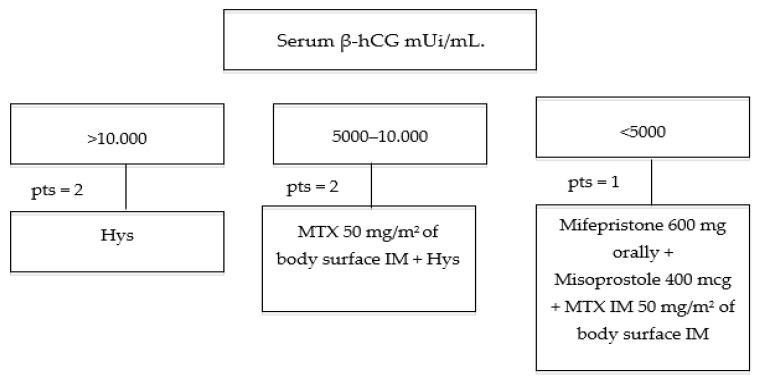
Management of cervical pregnancies on the basis of serum β-hCG levels. pts = number of patients.

The earlier ultrasound diagnosis of CP allows conservative procedures to preserve uterus in patient with desire for future pregnancies. With the increased incidence of IVF procedures, a resultant increase in CPs is expected. Moreover, the incidence of heterotopic pregnancy results higher in women who conceive after IVF treatments (1:30,000 vs. 1:100 pregnancies) [19]. On this basis we retain that conservative management of CP represents an emerging challenge. The best approach for this rare condition management is not yet available. This work is based on a small sample of cases but it allows to compare the impact of different fertility-sparing strategies for CPs. According to our cases, in haemodinamically stable patients with a diagnosis of CP at an early gestational age, serum βhCG levels and its indirect estimation of trophoblast development could guide in choosing the best conservative treatment. Our data suggest that medical therapy, with the oral administration of MTX, Mifepristone and Misoprostol, represents an effective option for the management of CP with serum βhCG levels < 5000 mUI/mL. Considering βhCG serum levels between 5000 and 10,000 mUI/mL, a combined approach using systemic IM injection MTX and hysteroscopy could be safety and effective. Hysteroscopy alone appears to be feasible for conservative management of CP if it is performed by an expert surgeon, also in case of high βhCG levels (>10,000 mUi/mL) (Figure 6). Total hysteroscopic approach in two steps, have to ensure pregnancy interruption, complete resection of the trophoblasts and prompt coagulation of the vessels in the implantation site. Furthermore, hysteroscopy, alone or in combination with MTX, may provide a greater effect on the descent of βhCG, leading to a reduction of the hospitalization stay, decreasing costs and period for attempt pregnancy. Further prospective studies on larger samples are needed to define therapeutic protocols for CP management.

## Figures and Tables

**Table 1:** Fertility-sparing management in cervical pregnancies: A single center experience.

Cases	GA (Weeks)	Basal βhCG mUi/ml	Management	βhCG mUi/mL after Procedure	Time until βhCG Undetectable < 5 mUi/mL (Days)	Outcomes
Case 1	6 + 6	55,951	1st step: Hys: CP resection by twizzle; 2nd step: Hys: bipolar resection of residual trophoblastic material	8822	20	Complete resolution
Case 2	6 + 6	10,862	1st step: Hys: CP resection by twizzle; IC Foley catheter 2nd step: Hys: vessels elettrocoagulation by bipolar twizzle	After 1st step: 6951 After 2nd step: 3171	40	Hemorrhage with blood loss 1400 cc and blood transfusion (after 1st step) Complete resolution After 5 months: spontaneous pregnancy with normal site of implantation
Case 3	6	4274	MTX IM 50 mg/m^2^ of body surface + Hys: CP resection by twizzle	886	15	Complete resolution
Case 4	5	9747	MTX IM 50 mg/m^2^ of body surface + Hys: CP resection by twizzle	2557	24	Complete resolution
Case 5	6 + 6	1331	Mifepristone 600 mg orally + Misoprostol 400 mcg + MTX IM 50 mg/m^2^ of body surface	1082	34	Complete resolution After 4 months TVUS: image in the cervical portion due to residual CP

CP: Cervical pregnancy, GA: Gestational age, MTX: Methotrexate, IM: Intramuscular, Hys: Hysteroscopy, IC: Intracervical, TVUS: Transvaginal ultrasounds.

**Table 2:** Risk factors profile.

Cases	Age	Risk Factors for CP
1	41	Previous CP Previous miscarriage in 1st trimester: RCU Previous late abortion with retained placental residues: RCU
2	36	Previous tubal EP LPS: myomectomy Onset of pregnancy: IVF
3	37	Previous miscarriage Onset of pregnancy: IVF
4	37	Smoke Onset of pregnancy: IVF
5	35	None

RCU: Curettage, EP: Ectopic pregnancy, IVF: In vitro fertilization.
